# Supplementary material for: Cyclic-di-GMP Induces STING-Dependent ILC2 to ILC1 Shift During Innate Type 2 Lung Inflammation
Source: Front Immunol. 2021 Feb 18;12:618807. doi: 10.3389/fimmu.2021.618807 (PMC7935536; doi:10.3389/fimmu.2021.618807)
Supplement: Supplementary file 1 [file Presentation_1.pdf]

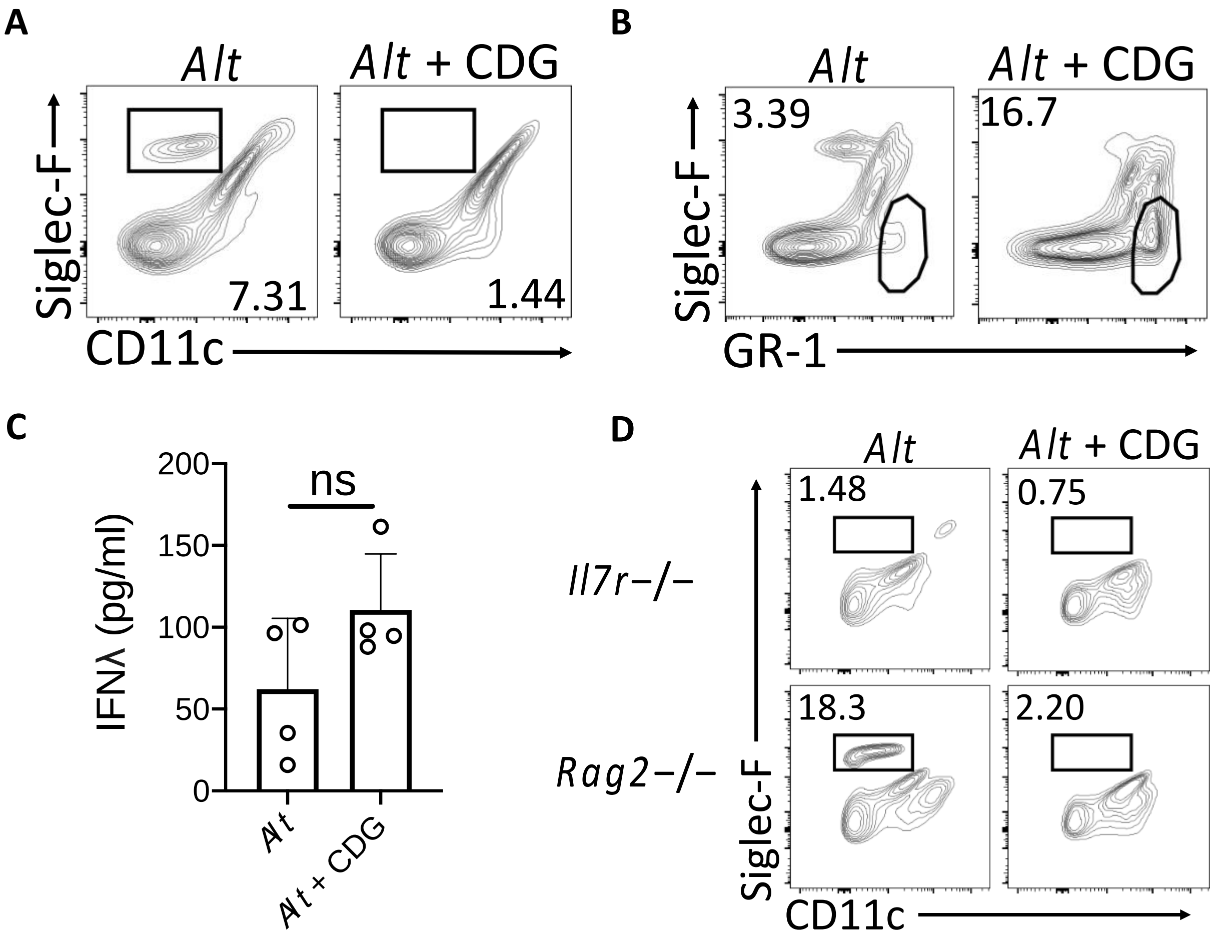

SUPPLEMENTARY FIGURE 1 | CDG abrogates *Alt*-induced type 2 inflammation and increases neutrophilia. Mice were challenged using the same model as in **Figure 1A**. Representative flow plots for eosinophil **(A)** and neutrophil **(B)** identification in wild type mice. **(C)** BAL IFNλ ELISA. **(D)** Representative flow plots for eosinophil identification in *Rag2*-/- and *Il7r*-/- mice. Data shown are representative of 2-10 independent experiments with 2-4 mice per group.

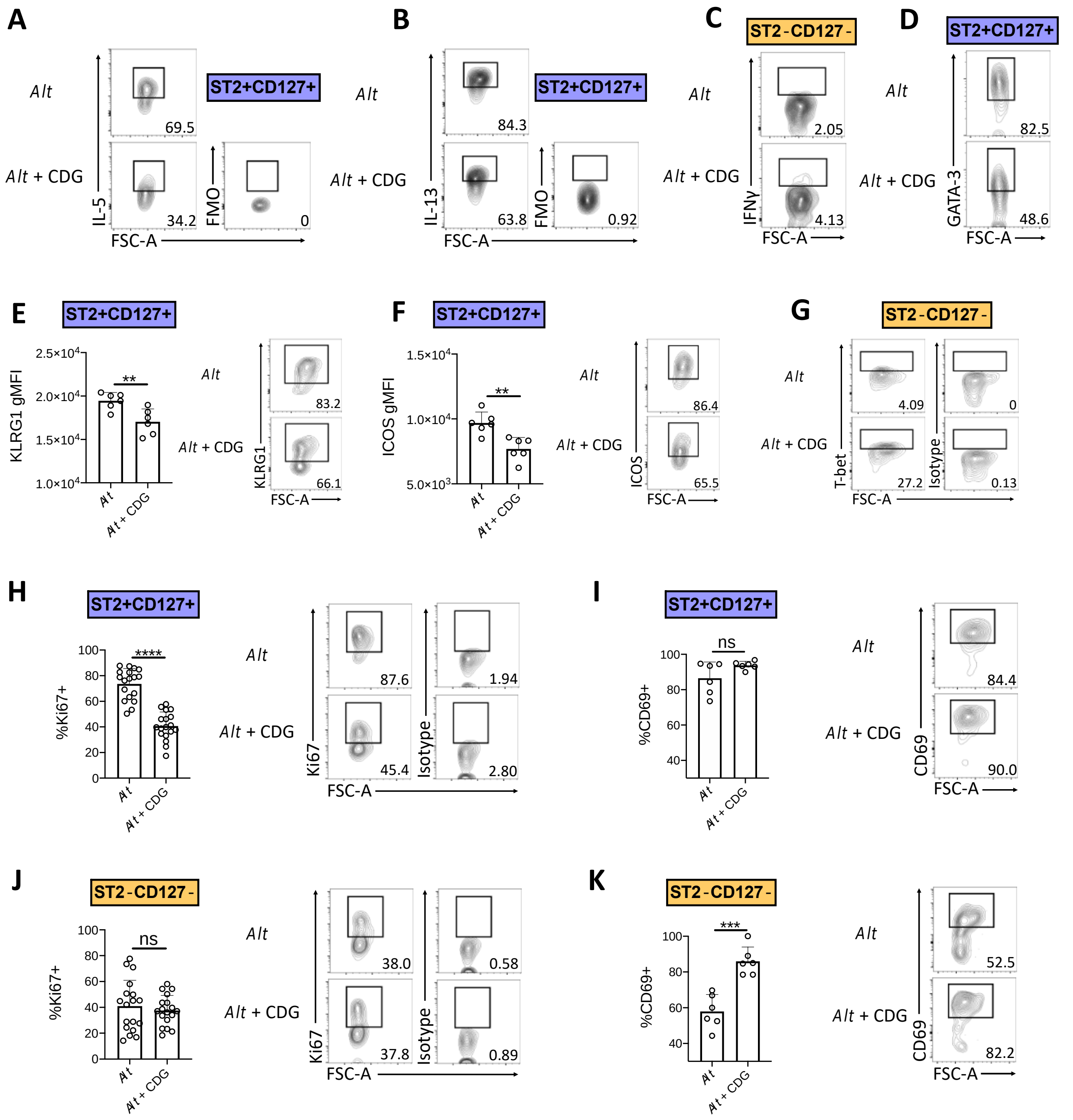

SUPPLEMENTARY FIGURE 2 | CDG induces innate lymphoid cell compartmental changes. Mice were challenged using the same model as in **Figure 1A**. Representative flow plots for lung ILC2 IL-5 **(A)**, ILC2 IL-13 **(B)**, ILC1 IFNγ **(C)** and ILC2 GATA-3 **(D)**. **(E)** Geometric mean fluorescent intensity (gMFI) of lung ILC2 KLRG1 (left) and representative flow plots (right). **(F)** gMFI of lung ILC2 ICOS (left) and representative flow plots (right). **(G)** Representative flow plots for lung ILC1 T-bet. **(H)** Frequency of Ki67+ lung ILC2s (left) and representative flow plots (right). **(I)** Frequency of CD69+ lung ILC2s (left) and representative flow plots (right). **(J)** Frequency of Ki67+ lung ILC1s (left) and representative flow plots (right). **(K)** Frequency of CD69+ lung ILC1s (left) and representative flow plots (right). Data shown are representative of 2-7 independent experiments with 2-4 mice per group. **P < .01, ***P < .001, ****P < .0001 unpaired t test.

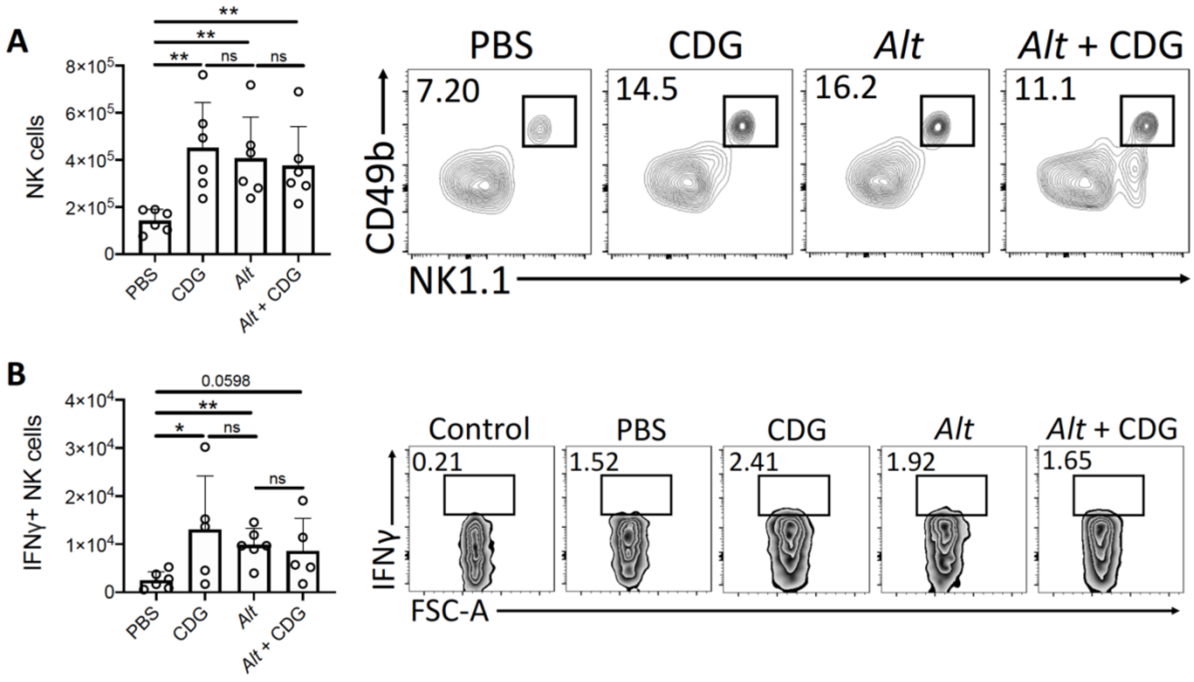

SUPPLEMENTARY FIGURE 3 | CDG does not potentiate *Alt* induced NK cell accumulation. Mice were challenged using the same model as in **Figure 1A**. **(A)** Total lung NK cells (left) and representative flow plots (right). **(B)** Total IFNγ+ lung NK cells (left) and representative flow plots (right). Data shown are representative of 2 independent experiments with 2-3 mice per group. *P < .05, **P < .01, unpaired t test

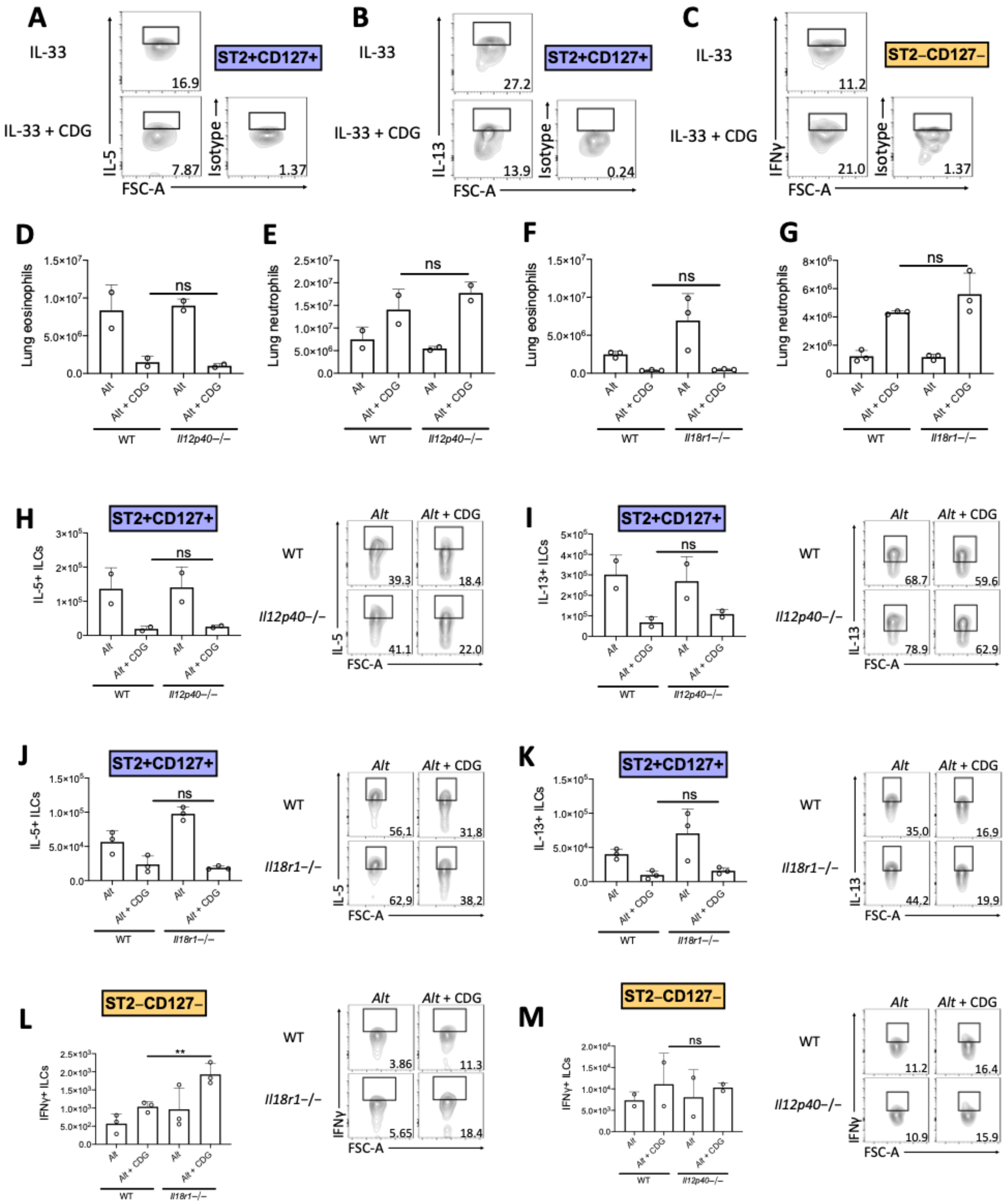

SUPPLEMENTARY FIGURE 4 | Suppression of type 2 inflammation occurs downstream of IL-33 and independent of IL-12 and IL-18 signaling. **(A-C)** Mice were challenged using the same model as in **Figure 1A** but with IL-33 instead of *Alt*. Representative flow plots for lung ILC2 IL-5 **(A)**, ILC2 IL-13 **(B)**, and ILC1 IFNγ **(C)**. **(D-G)** Mice were challenged using the same model as in **Figure 1A**. Total number of lung eosinophils **(D)** and neutrophils **(E)** in wild type and *Il12p40*-/- mice. Total number of lung eosinophils **(F)** and neutrophils **(G)** in wild type and *Il18r1*-/- mice. Total number of IL-5+ and IL-13+ ILC2s in *Il12p40*-/- **(H, I)** and *Il18r1*-/- mice **(J, K)**. Total number of IFNγ+ ILC1s in *Il18r1*-/- and *Il12p40*-/- mice **(L, M)**. Data shown are representative of 2 independent experiments with 2-4 mice per group. **P < .01, unpaired t test.

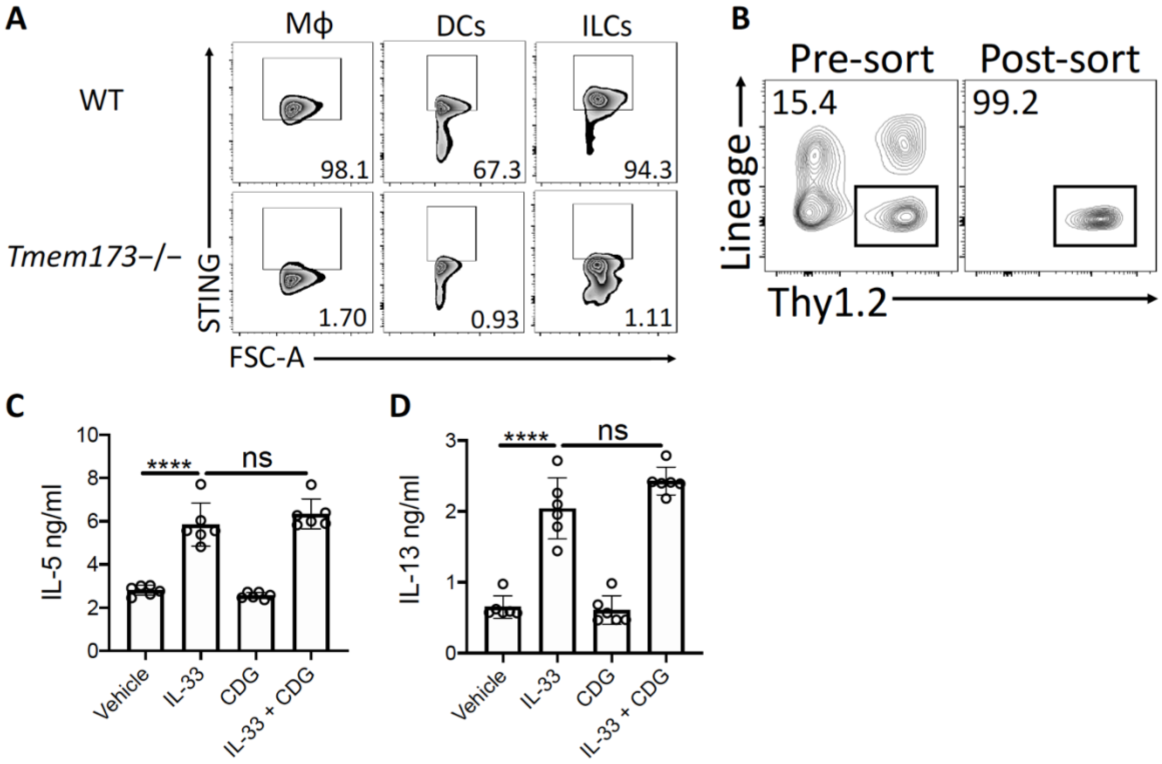

SUPPLEMENTARY FIGURE 5 | STING is expressed by macrophages, DCs, and ILCs and c-di-GMP does not suppress ILC2 activation *in vitro*. **(A)** BAL macrophage (Mφ), BAL dendritic cell (DC), and lung ILC STING expression **(B-D)** Sort purified ILCs were allowed to rest *in vitro* for 48 hours with 10ng/ml IL-2 and IL-7. Following media change, ILCs were cultured with either IL-2 (10ng/ml) and IL-7 (10ng/ml); IL-2 (10ng/ml), IL-7 (10ng/ml), CDG (10μM); IL-2 (10ng/ml), IL-7 (10ng/ml), and IL-33 (30ng/ml); or IL-2 (10ng/ml), IL-7 (10ng/ml), IL-33 (30ng/ml), and CDG (10μM). After 24 hours of stimulation, supernatants were collected for ELISA. **(B)** ILC sort purity. Supernatant IL-5 **(C)** and IL-13 **(D)**. ****P < .0001, unpaired t test.

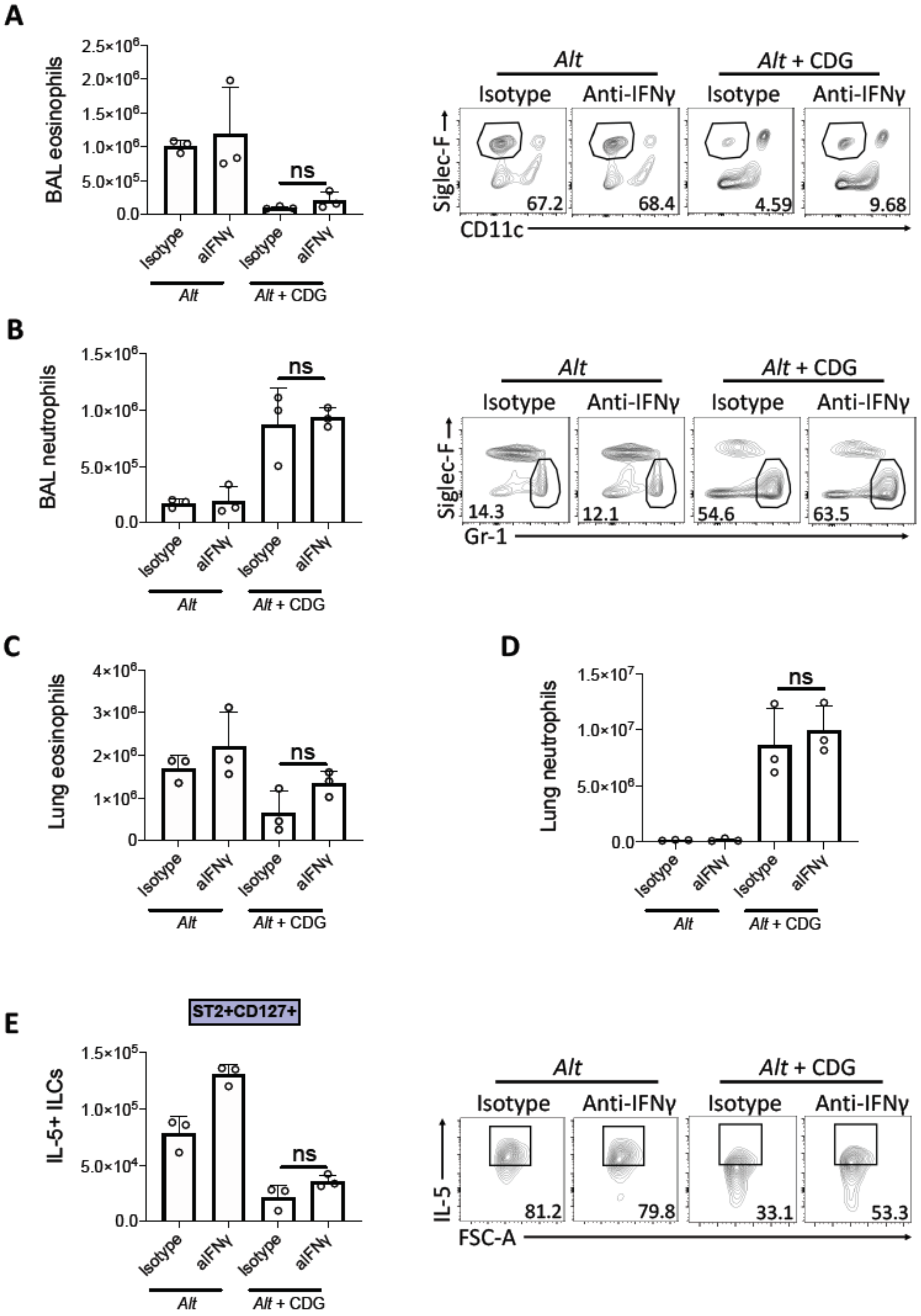

SUPPLEMENTARY FIGURE 6 | Intraperitoneal IFNγ blocking antibody (333ug, BioXCell) or control antibody injections were administered on D-1, D0, and D1 to mice undergoing the protocol in **Figure 1A**. **(A)** Total number of BAL eosinophils (left) and representative plots (right). **(B)** Total number of BAL neutrophils (left) and representative plots (right). Total number of lung eosinophils **(C)** and neutrophils **(D)**. **(E)** Total number of lung IL-5+ ILC2s.
